# Lack of Sik1 in Mouse Embryonic Stem Cells Impairs Cardiomyogenesis by Down-Regulating the Cyclin-Dependent Kinase Inhibitor p57^kip2^

**DOI:** 10.1371/journal.pone.0009029

**Published:** 2010-02-03

**Authors:** Antonio Romito, Enza Lonardo, Guglielmo Roma, Gabriella Minchiotti, Andrea Ballabio, Gilda Cobellis

**Affiliations:** 1 Telethon Institute of Genetics and Medicine (TIGEM), Naples, Italy; 2 Stem Cell Fate Laboratory, Institute of Genetics and Biophysics, Consiglio Nazionale delle Ricerche (CNR), Naples, Italy; 3 Centre for Genomic Regulation (CRG), Pompeu Fabra University (UPF), Barcelona, Spain; 4 Dipartimento di Patologia Generale, Seconda Università di Napoli, Naples, Italy; University of Giessen Lung Center, Germany

## Abstract

Sik1 (salt inducible kinase 1) is a serine/threonine kinase that belongs to the stress- and energy-sensing AMP-activated protein kinase family. During murine embryogenesis, *sik1* marks the monolayer of future myocardial cells that will populate first the primitive ventricle, and later the primitive atrium suggesting its involvement in cardiac cell differentiation and/or heart development. Despite that observation, the involvement of *sik1* in cardiac differentiation is still unknown. We examined the sik1 function during cardiomyocyte differentiation using the ES-derived embryoid bodies. We produced a null embryonic stem cell using a gene-trap cell line carrying an insertion in the *sik1* locus. In absence of the sik1 protein, the temporal appearance of cardiomyocytes is delayed. Expression profile analysis revealed *sik1* as part of a genetic network that controls the cell cycle, where the cyclin-dependent kinase inhibitor p57^Kip2^ is directly involved. Collectively, we provided evidence that sik1-mediated effects are specific for cardiomyogenesis regulating cardiomyoblast cell cycle exit toward terminal differentiation.

## Introduction

The formation of the heart involves a precisely orchestrated series of molecular and morphogenetic events, and even a subtle perturbation of this process can have catastrophic consequences for cardiac function. The specification of the appropriate numbers and types of cardiac cells is an early event during embryogenesis. These cells then migrate to form a simple, yet functional, heart tube. Further morphogenesis transforms this heart tube into morphologically and functionally discrete cardiac chambers [1].

Cardiomyogenesis depends on the regulated activities of numerous specific transcription factor genes, which encode members of the zinc finger [2], homeodomain [3], T-box [4], bHLH [5] and MADS domain families [6]. These factors act in a combinatorial way to create a positive feed-forward regulatory circuitry that controls the development of cardiac myocytes. A concomitant regulation in the expression and activities of cell-cycle regulatory molecules (cyclins, cyclin-dependent kinases and cyclin-dependent kinase inhibitors [CDKIs]) is essential for the control of cell proliferation that is concurrent with differentiation [7]. Among multiple cell-cycle regulators, CDKI p57^Kip2^ is the first to be detectable in the developing heart, at E10.5, and is involved in cardiac cell-cycle exit during chamber maturation [8].

The sik1 protein was identified in a screen for kinases specifically expressed in the heart of the mouse embryo [9]. During mouse embryogenesis, *sik1* expression is detected at 8.0 d.p.c. in the monolayer of the future myocardial cells; it is rapidly down-regulated at 8.5 d.p.c. upon formation of the primitive ventricle, although it is still present in the myocardial cells that will populate the primitive atrium and bulbus cordis. At 9.5 d.p.c. *sik1* expression is down-regulated in the primitive atrium but still detected in the sinus venosus and truncus arteriosus. The expression pattern of *sik1* gene suggests a role during the earliest stages of myocardial cell differentiation and/or primitive chamber formation [10]. Recent studies have demonstrated that sik1 protein phosphorylates class II HDACs *in vivo*, triggering the cytoplasmic export of the HDACs and activation of MEF2-dependent transcription [11], [12].

The specific expression pattern of *sik1* during mouse development prompted us to investigate the role of sik1 in the regulation of cardiac lineage commitment in a stem-cell model system. Embryonic stem (ES) cells can differentiate into derivatives of all three of the primary germ-cell layers, including cardiomyocytes, and previous studies have suggested that early steps in embryonic cardiomyogenesis take place during embryoid body (EB) differentiation of ES cells [13]. Using an ES cell line carrying a gene-trap insertion in the *sik1* gene, we produced a *sik1*-deficient cell line. The cardiac developmental program in the ES cell/EB model is affected in the absence of sik1. The number of beating colonies was significantly decreased in sik1^flp/flp^ EBs and analysis of cardiac markers revealed a down-regulation of terminal cardiac markers (*αMyHC, Mlc2v, aCach* and *cTnI*). When analyzed in depth, these cells showed a significant delay in cardiac differentiation in the absence of sik1, due to a defect in cell cycle exit of differentiating cardiomyoblasts. Gene profiling studies were performed to identify genes that may play a role in regulating *sik* specific function. One of the most down-regulated genes with cardiac expression was *cdkn1c* codifying for p57^Kip2^, a cyclin-dependent kinase inhibitor (CDKI), which showed a peculiar transcriptional regulation during cardiac differentiation, lost in the absence of *sik1*.

Forced expression of p57^Kip2^ in *sik1*-deficient ESCs was able to rescue the mutant phenotype.

Our data thus indicate that *sik1* via p57^Kip2^ might have a central role in the control of the exit of cardiomyoblasts from the cell cycle toward the terminal differentiation of cardiomyocytes.

## Results

### Generation of sik1^flp/flp^ ES Cell Clone

**Figure 1:**
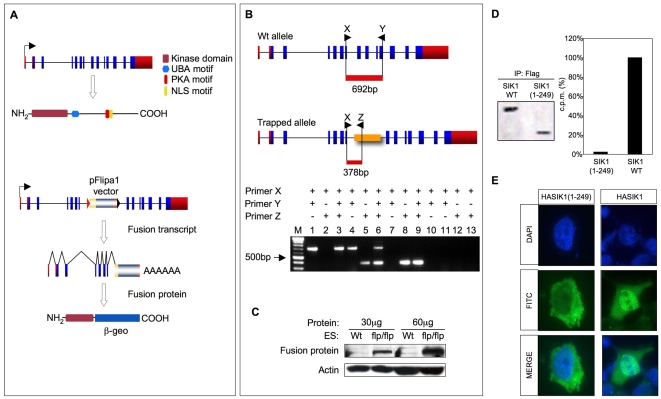
Effects of gene trapping insertion and screening of homozygous mutant ES cells. (**A**) Schematic representation of the *sik1* gene encoding for a protein of 779 amino acids. The SIK1 protein structure is visualized: the kinase domain (27 to 278), the UBA motif (303–343), the PKA motif (567–584) and the nuclear localization signal (586 to 612). The insertion of the gene-trap vector occurred in the seventh intron of the wild-type *sik1* gene, causing a deletion of the protein segment from residue 250 to 779. The corresponding fusion transcript and the resulting protein are depicted. (**B**) Identification of homozygous ES clones for the trapped allele with RT-PCR analysis. Schematic representation of the oligonucleotides used to discriminate between the wild-type and fusion transcripts. X, Y and Z correspond to primers sik1UP, sik1LW and L232, respectively. The amplification of wild-type and mutant cDNA results in two products of different molecular weight, 682 bp and 378 bp respectively. Lanes: 1,2 and 3, amplification of *sik1^wt/wt^* cDNA; 4,5 and 6, amplification of *sik1^wt/flp^* cDNA; 7,8 and 9 amplification of *sik1^flp/flp^* cDNA; 10, *sik1^wt/wt^* total RNA; 11 and 12, *sik1^wt/flp^* total RNA; 13, *sik1^flp/flp^* total RNA. The arrow indicates the 500-bp band of the DNA molecular weight marker. The arrow indicates the 500-bp band of the DNA molecular weight marker. (**C**) Western blot on proteins extracted from wild-type and *sik1^flp/flp^* ES cells using an antibody against the β-galactosidase. In *sik1^flp/flp^*ES cells is possible to detect a band corresponding to the fusion protein that is absent in wild-type ES cells. Different amounts of proteins were used on western blot. (**D**) Expression plasmids (6 µg) for FLAG-tagged sik1 and its truncated version (1–249aa) were transfected into HEK293T cells and subjected to immunoprecipitation with FLAG-M2 resin. Aliquots of FLAG-SIK1 wt and truncated IPs were subjected to western blotting (WB) using an anti-FLAG antibody (left panel) and to in vitro kinase assays using [γ-^32^P]- ATP with AMARA peptide as a substrate (right panel). The relative activation was calculated by subtracting counts incorporated in the assay of non-immune sample from gross counts of immunoprecipitated samples to determine net cpm. (**E**) Intracellular localization of HA-tagged wild-type and mutant SIK1 proteins in HEK293T cells.

To study the role of the *sik1* gene during cardiomyocyte differentiation, we used a gene-trap ES cell line (GC389) (http://genetrap.tigem.it/public) [14] carrying a pFlipa1 vector insertion, as shown in figure 1. The fusion transcript generated by the gene-trap vector insertion directs the expression of a truncated protein carrying only the N-terminal domain of SIK1 (residues 1 to 249) fused to the β-geo cassette (Fig. 1A).

We mutated the second allele of *sik1* by cultivating heterozygous mutant cells (*sik1^wt/flp^*) in increasing concentrations of the neomycin analogue G418, as previously described [15]. Several independent surviving clones were isolated and analyzed by RT-PCR to discriminate cells that were homozygous for the trapped allele (*sik1^flp/flp^*) over *sik1^wt/flp^* cells. Clones carrying the gene trap insertion on both alleles that did not express the wild-type transcript anymore were identified (Fig. 1B, lanes 7, 8 and 9). The presence of the fusion protein in *sik1^flp/flp^* ES cells was confirmed by western blot analysis using an antibody against the β-galactosidase (Fig. 1C). Moreover, enzyme activity, determined as the ability of truncated sik1 to phosphorylate the specific AMARA peptide substrate in comparison to the full-length protein, was measured. To this end, FLAG-tagged sik1 (1–249) and full-length protein were expressed in HEK293 cells and, after immunoprecipitation, the peptide phosphorylation activity was measured using radio-labeled ATP. As expected, the full-length sik1 was able to efficiently phosphorylate the AMARA substrate; whereas the phosphorylation signal of the mutant protein was dramatically reduced, indicating that the catalytic activity of the truncated sik1 is almost inactive (Fig. 1D). We then evaluated the subcellular localization of the truncated form of sik1 (residues 1 to 249) produced by the trapped allele. It is known that the wild-type protein is resident in the nucleus where it exerts its function [16]. In contrast, the cells expressing the truncated form of sik1 showed an intense signal only in the cytoplasmic compartment, as opposed to the full-length protein that was mainly located in the nucleus (Fig. 1E). The gene-trap insertion resulted in the deletion of the nuclear localization signal; therefore, as expected, this truncated version of the sik1 protein showed a different localization compared to the full-length sik1.

Altogether, these data indicate that the gene-trap insertion interrupts sik1 expression and results in the production of an inactive protein with a cytoplasmic localization.

### Delayed Cardiomyocyte Differentiation in sik1^flp/flp^ ES Cells

**Figure 2:**
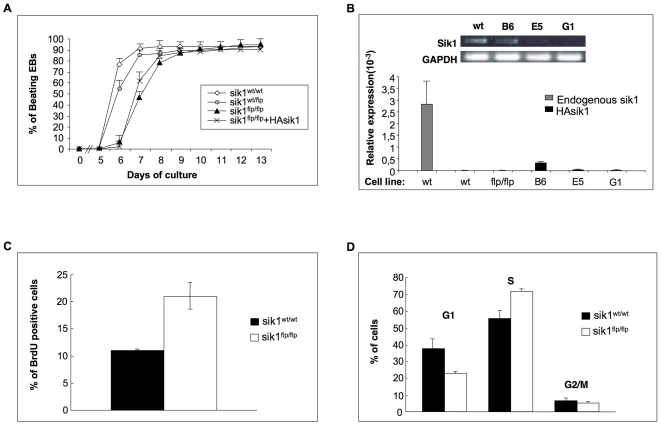
*In vitro* differentiation and proliferation potential of *sik1^flp/flp^* ES cells. (**A**) Percentages of EBs differentiating to beating cardiomyocytes. EBs derived from wild-type, *sik1^wt/flp^*, *sik1^flp/flp^* and *sik1^flp/flp^*+HA-sik1 ES cells were used to monitor differentiation into cardiomyocytes. *Sik1^flp/flp^* + HAsik1 used corresponds to HAsik1 B6 clone expressing the highest level of sik1 (**B**). Three independent experiments were performed, each plating an equal number of EBs (n = 120). The statistical significance of the observed differences between the percentage of wild-type and mutant beating EBs were analyzed and the time points in which major differences were observed (days 6 and 7) showed significant p-values of 1.109e^−08^ and 3.271e^−08^, respectively. P-values were calculated using a two-sample test for equality of proportions with continuity correction. (**B**) RT- and Q-PCR analysis on wild-type ES cells and three representative clones carrying the HA-sik1 transgene. Data represent fold change of transgene expression vs. sik1 endogenous level. (**C**) Proliferation assay measured by BrdU incorporation. (**D**). Cell-cycle distribution of the wild-type and sik1^flp/flp^ populations by FACS analysis.

The next step was to evaluate the developmental potential of *sik1^flp/flp^* ES cells to differentiate in cardiomyocytes compared to wild-type and *sik1^+/flp^* cells using the “hanging drop” method. The embryoid bodies (EBs) generated clusters of adherent cells that began to contract rhythmically, indicating differentiation to the cardiac lineage, a derivative of the mesoderm layer. Seventy-six percent of plated EBs derived from wild-type ES cells showed areas of contracting cardiomyocytes starting from the day after adhesion (day 6, Fig. 2A). The appearance of beating EBs increased and reached over 92% at day 8 of culture. In contrast, EBs derived from *sik1^flp/flp^* cells showed a dramatic reduction in the formation of contractile areas (5% at day 6). However, a gradual increase of beating EBs was seen in the following 4 days of culture, when the *sik1^flp/flp^* EBs reached the same percentage of beating EBs as wild-type cells (Fig. 2A). The EBs derived from *sik1^+/flp^* cells showed an intermediate phenotype compared to *sik1^flp/flp^* EBs, which can be attributed to a gene-dosage effect.

We then tested whether functional rescue of cardiomyocyte differentiation can be achieved by gene re-introduction. To this purpose, we generated stable clones overexpressing the HA-tagged *sik1* transgene. Several drug-resistant clones were amplified and RNA extracted to measure the expression of HA-sik1 by qPCR. Sik1 transgene expression was detected in all clones analyzed; however, none of them reached the basal level of endogenous *sik1 expression* (Fig. 2B). The clone B6, which showed the highest level of transgene expression, was induced to differentiate into cardiomyocytes. The *HA*-*sik1*(B6) overexpressing clone only partially restored the appearance of the beating cardiomyocytes (Fig. 2A). Since over-expression experiments do not allow the modulation of kinase activity both in terms of timing and signal strength and, moreover, since the levels of transgene expression in selected clones were lower than in the heterozygous cells, the combination of these drawbacks might explain the incomplete rescue (Fig. 2A).

Taken together, these data indicate that the *sik1^flp/flp^* population can differentiate into contracting cardiomyocytes, but showing a consistent delay in cardiomyogenesis.

Having shown that lack of *sik1* led to a delayed differentiation, we assessed the proliferative potential of *sik1* ^flp/flp^ ES cells by BrdU incorporation, postulating that the lack of *sik1* might increase the proliferation rate of these cells. In line with this hypothesis, the rate of *sik1* ^flp/flp^ cell proliferation significantly increased (15%) compared to the wild-type cells (Fig. 2C). These data were also confirmed by cell counting (data not shown). The increased proliferation rate of *sik1* ^flp/flp^ ES cells resulted from a shortened G1 phase (*wt* 37% *vs*. *sik1* ^flp/flp^ 23%, Fig. 2D) and accumulation in S-phase, (wt 55% vs. *sik1* ^flp/flp^ 72%, Fig. 2D).

### sik1 Deficiency Specifically Affects Cardiac Differentiation of ES Cells

**Figure 3:**
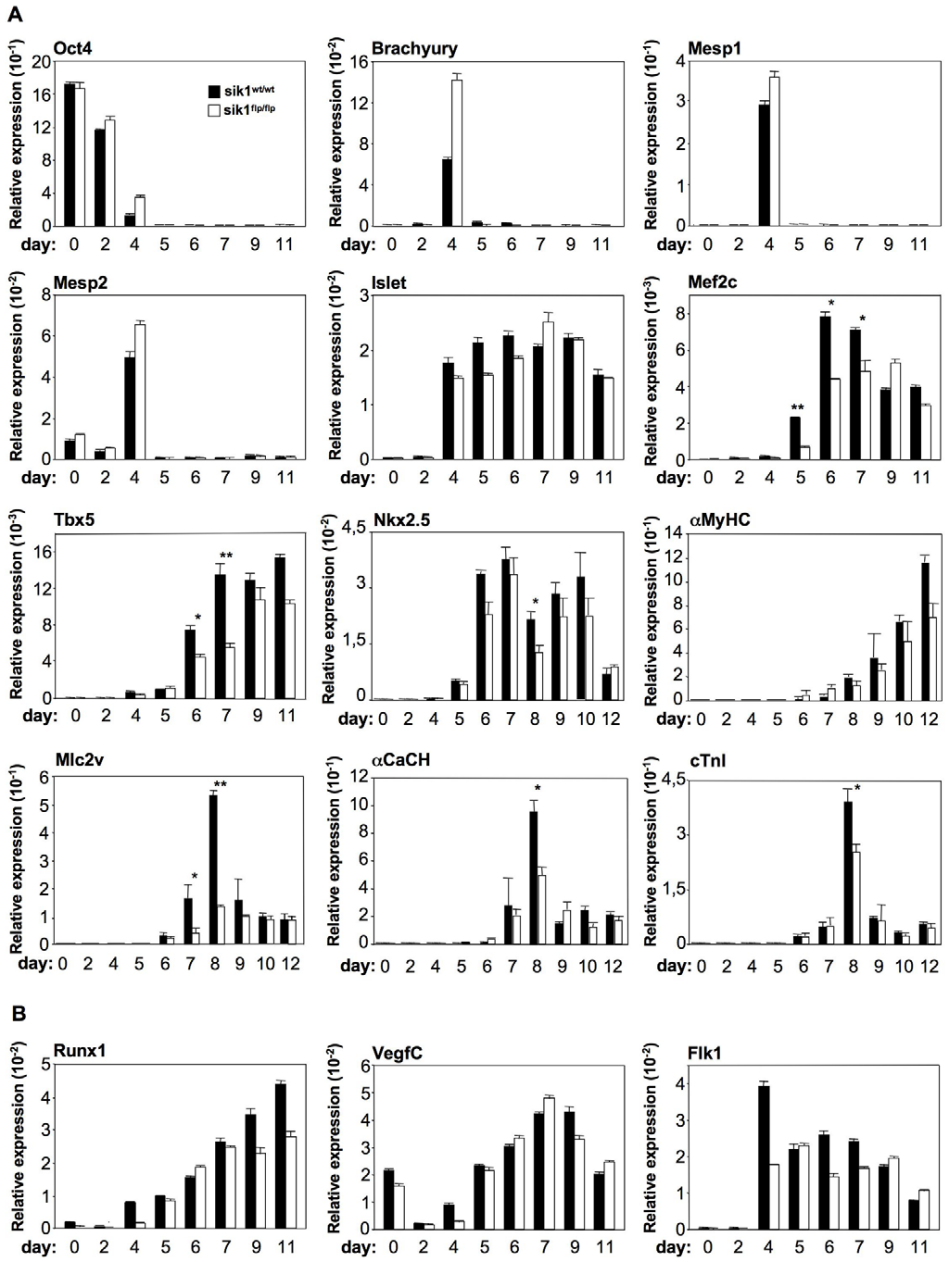
sik1 deficiency affects terminal cardiogenic differentiation. (**A**) Q-PCR of a pluripotency gene (*Oct3/4*), of an early mesodermal marker (*brachyury*), of pre-cardiac mesodermal markers (*mesp1*, *mesp2, Isl1*), early cardiogenic markers (*Mef2c, Tbx5, Nkx2.5*) and terminal cardiac markers (*α-MyHC*, *Mlc2V*, *αCach* and c*TnI*) during *in vitro* differentiation of wild-type and *sik1^flpflp^* ES cells. (**B**) Q-PCR on hematopoietic/endothelial markers (*runx1*, *vegfC* and *flk1*). n = 3, *, P<0.05, **, P<0.001 vs. control cells. P values were calculated using a two-tailed, unpaired *t* test.

We evaluated the expression of pluripotency genes as well as of mesoderm induction and specification markers. We measured the expression of *Oct-3/4* transcript, a pluripotency marker, and *Brachyury* transcript, which is required for correct mesoderm formation and patterning [17]. As shown in figure 3A, *sik1* deficiency did not affect *Oct-3/4* expression, which was progressively downregulated in both cell populations, nor *Brachyury*, which was nearly absent in the undifferentiated ES cells and transiently expressed during embryoid body formation peaking at day 4. At the same time, committed mesodermal cells started to express similar levels of pre-cardiac mesodermal genes (*Mesp1*, *Mesp2* and *Islet-1* [18] in both the wild-type and in the *sik1^flp/flp^* differentiating populations.

Given the delay in cardiomyogenesis (Fig. 2A), we studied cardiac differentiation in *sik1^flp^* ^/flp^ differentiating EBs measuring the levels of cardiogenic transcription factors (*Nkx2.5, Tbx5 and Mef2C*), which are first expressed in cardiomyoblast cells [19]. These factors then act in combination to activate the expression of several cardiac structural genes, i.e. myosin heavy chain (*α-MyHC*), myosin light chain (*Mlc2V*), calcium channel (*αCach*) and cardiac troponin (c*TnI*) [20]. We measured the expression of these genes both in the wild-type and in the *sik1^flp/flp^* differentiating EBs. *Nkx2.5*, *Tbx5* and *Mef2C* expression started at day 5 in the wild-type EBs and progressively increased. In the *sik1^flp/flp^* differentiating EBs, the expression of these genes was impaired in this time window; in line with the morphological analysis, the expression of the cardiac structural genes (*α-MyHC, Mlc2V, αCach* and c*TnI*) was impaired from day 6 to day 8, whereas it increased from day 9 onward to reach levels comparable to wt cells. (Fig. 3A),

Next, we also examined the influence of *sik1* on the induction of the hematopoietic/endothelial lineage, measuring the expression of *Runx1*, *VegfC* and *Flk1*. As shown in figure 3B, the levels of expression of these genes were not significantly impaired in the *sik1^flp/flp^* differentiating population.

Altogether, these data suggested that mesoderm formation and patterning were not altered *ab inicio*, as demonstrated by the correct expression of *Mesp1*, *Mesp2* and *Isl1*, but the cardiogenic programme of these cells was delayed, as showed by the downregulation of *Nkx2.5*, *Tbx5* and *Mef2C* genes as well as of markers of terminal cardiac differentiation (*αMyHC, Mlc2v, αCach, cTnI*).

**Figure 4:**
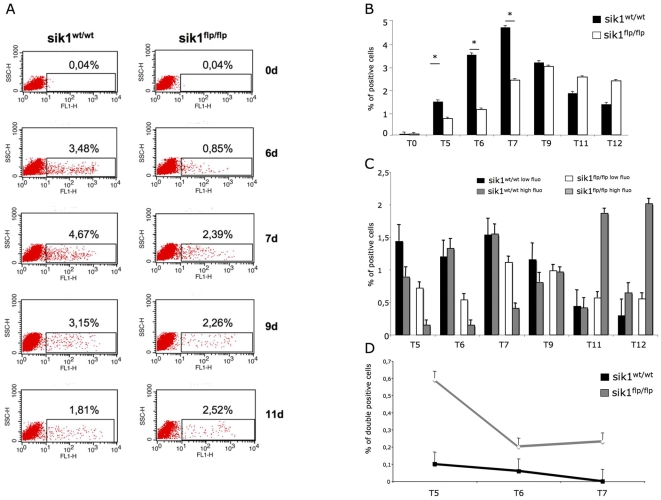
Measurement of myosin positive cells in wild-type and *sik1^flp/flp^* EBs by FACS. (**A**) EBs were analyzed by FACS at days 0, 5, 6, 7, 9, 11 and 12 of differentiation with the antibody MF20. Data represent results of one out of three independent experiments. (**B**) Graphic representation of FACS analysis. Data represent mean ± S.E. of three biologically independent experiments (*, p<0.05 compared with wild-type EBs. P-values were calculated using a two-tailed, unpaired *t* test). (**C**) Graphic representation of low fluorescence vs high fluorescence MF20 positive cells distribution, as by FACS analysis (n = 3). Wild-type and *sik1^flpflp^* cells were used throughout the analysis. (**D**) Costaining experiment using anti-troponin and anti-BrdU to detect proliferating cells only in cardiomyocytes population.

In order to ascertain whether lack of *sik1* affected the number of cardiac cells, we quantified the percentage of cardiomyocytes derived from wild-type and *sik1^flp/flp^* EBs throughout the differentiation by FACS analysis. To this end, we used the sarcomeric myosin (MF-20) staining (Fig. 4A, 4B). In line with our hypothesis, from day 5 to day 7, the *sik1^flp/flp^* -derived EBs showed a reduced number of MF-20 positive cells, compared to the *wt* EBs. However, at day 9, the number of cardiomyocytes was fully rescued in the *sik1^flp/flp^* EBs, according to the morphological and qPCR analysis reported above (Fig. 2A–3A).

Furthermore, we analyzed the FACS data by discriminating high fluorescence (hf) vs. low fluorescence (lf) cells expressing sarcomeric myosin (MF-20). Remarkably, while a significant number of hf cells already appeared at d5 in wt EBs and the hf vs. lf ratio was comparable throughout the differentiation, in *sik1^flp/flp^* EBs, consistent number of hf cells can be detected only starting from day 9 onward (Fig. 4C).

All together, these data supported the idea that the appearance of cardiomyocytes in *sik1^flp/flp^* EBs occurred as a slower wave of differentiation.

Worth noting, in many cell types the differentiation is strictly dependent on the cell cycle withdrawal. We thus reasoned that the difference observed in the two cell populations may be due to a defect of the mechanism that regulates cell cycle exit toward differentiation of cardiomyocytes. To directly address this issue, we carried out FACS analysis to study the concurrent cell cycle withdrawal and differentiation in the cardiomyocyte population in both groups and in the most critical days of differentiation (d5–d7). To this end, we performed double immunostaining studies using an antibody against a cardiac sarcomeric protein (cTnI) coupled with the analysis of cell cycle activity (BrdU). On day 5, double positive cells dramatically increased in *sik1^flp/flp^* vs. wt population, indicating that cell cycle exit of cardiomyocytes was impaired in absence of sik1 (Fig. 4C). Furthermore, at day 6, few double-positive cells were detected in the wt population, which almost disappeared at day 7. On the contrary, in the *sik1^flp/flp^* population, the MF20/BrdU-positive cells decreased in number at day 6 but were still present at day 7, supporting the idea of a delayed cell cycle withdrawal in absence of sik1.

### Lack of sik1 Does Not Affect Neuronal Differentiation

**Figure 5:**
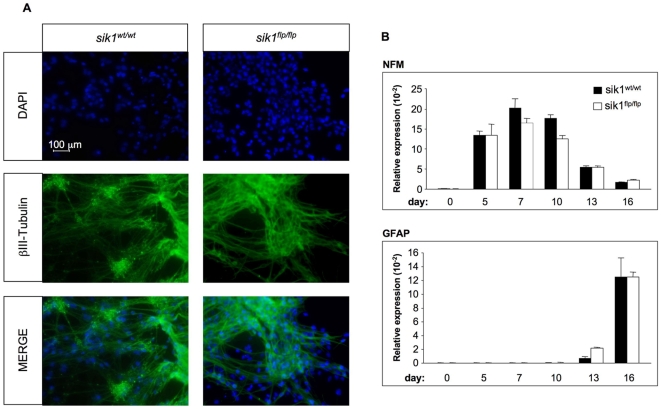
Neuronal differentiation of wild-type and *sik1^flp/flp^* ES cells. (**A**) Immunofluorescence analysis of wild-type and *sik1^flp/flp^* EBs at day 16 of differentiation using an anti-βIII-tubulin antibody reveals the presence of neurons. (**B**) Quantitative real-time PCR analysis to measure the expression of the neuron- and glial-specific markers *NFM* and *GFAP*, respectively, in wild-type and *sik1^flp/flp^* EBs at different time points during differentiation, n = 3.

In order to exclude a general effect of sik1 deficiency on ESC differentiation, we evaluated whether *sik1* deficiency impairs neuronal differentiation. As shown in figure 5A, both the wild-type and the *sik1^flp/flp^* EBs were able to generate a dense network of neurite growth [21]. The neural nature of these cells was confirmed by staining with an antibody against the neuron-specific form of class βIII-tubulin (Fig. 5A). We also measured the expression of the neuron- and glia-specific markers *NFM* and *GFAP* (Fig. 5B). The expression of *NFM* and *GFAP* was similar in the wild-type and *sik1^flp/flp^* populations, thus indicating that the EBs carrying the *sik1* mutant alleles have the same competence to acquire the neural phenotype as the wild-type EBs; supporting the idea of a cardiac specific effect of sik1 deficiency.

### The p57^Kip2^ Transcript Is Strongly Downregulated in sik1^flp/flp^ ES Cells

Given the role of sik1 as transcription regulator [12], we studied the transcriptome influence of sik1 in ES cells. We decided to perform the experiment on undifferentiated ES cells, comparing wild-type and *sik1^flp/flp^* cells, for three reasons: 1. to consider a homogenous cell population compared to differentiating EBs, which are known to contain different cell types; 2. to avoid an enrichment of cardiac specific genes, already found to be significantly altered in the previous analysis, and 3. to possibly reveal an early transcriptional event that may play a role in embryonic development. Using the filtering criteria of a two-or-greater-fold change in expression and a false discovery rate of less than 5%, 448 out of over 22,000 transcripts were differentially expressed. Under these conditions, there were 172 transcripts with increased expression in the *sik1^flp/flp^* ES cells, while 276 transcripts showed decreased expression (Table S1 and S2). To identify the biological processes in which the genes transcriptionally affected in *sik1^flp/flp^* ES cells are involved, we performed a Gene Ontology analysis. The analysis revealed a significant enrichment in different GO Biological Process categories, such as “steroid metabolic process”, “calcium ion transport”, “ion transport” and “di-, tri-valent inorganic cation transport” (Table S3) in which *sik1* is already known to be involved [16], [22]. This observation suggests that *sik1* deficiency has an effect on the transcriptome in ES cells, which is consistent with the data obtained.

Sik1 is known to regulate transcription through inhibitory phosphorylation of a family of CREB coactivators, called TORCs/CRTCs, and that down-regulation of *sik1* would be expected to increase the expression of CREB target genes [23] As further analysis, we decided to investigate whether genes found to be differentially expressed in the *sik1^flp/flp^* ES cells could be under the regulation of transcriptional factors belonging to the CREB family.

Interestingly, Table S4 shows that CREB transcription binding sites are found to be over-represented in upregulated genes, while they are clearly under-represented in the downregulated genes.

Given these data, which provide additional evidence of sik1 deficiency activity in our cells, we further analyzed the data obtained by array experiments using two stringent criteria: 1. looking for genes expressed in the heart; 2. looking for genes already known to cause cardiac phenotype when mutated (i.e. knock-out models). Using the MGI (mouse genome informatics) database, we identified 68 genes (42 downregulated and 26 upregulated) among the transcriptionally affected genes (Table S5). Interestingly, one of the most downregulated genes expressed in the heart is *cdkn1c* (−2.75 (log2); p-value, 1.11E-16). *Cdkn1c* gene codifies for the p57 protein belonging to the family of KIP/CIP factors that are cyclin-dependent kinase inhibitors (CDKI) involved in cell cycle arrest.

### Sik1 Regulates p57kip2 Only in the Cardiogenic Program

It is worth noting here that the levels of p57^Kip2^ are very low in proliferating cells and differentiation signals result in its accumulation, which is necessary to withdraw cells from the cell cycle and to move them toward differentiation [7]. Given that p57^Kip2^ is involved in regulating the cell cycle, it is possible to reason that it could be involved in the sik1 deficiency -dependent delayed cardiomyocyte differentiation.

**Figure 6:**
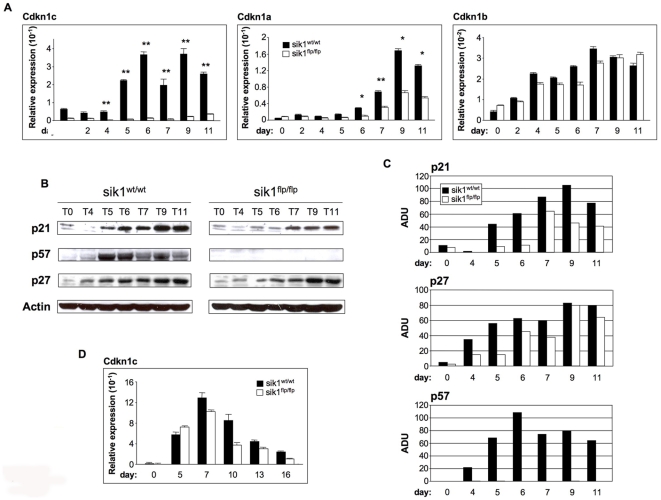
Expression profile of CDKIs during both cardiomyocyte and neuron differentiation. (**A**) Expression profile of genes encoding for CDK inhibitors belonging to the KIP/CIP family (*Cdkn1a*, *Cdkn1b* and *Cdkn1c*) measured by quantitative real-time PCR at different time points during cardiomyocyte differentiation of wild-type and *sik1^flpflp^* ES cells, n = 3, *, P<0.05, **, P<0.001 vs. control cells. P values were calculated using a two-tailed, unpaired *t* test. (**B**) Western blots analysis of CDKIs protein level during *in vitro* cardiomyocyte differentiation of wild-type and *sik1^flpflp^* ES cells. (**C**) p21, p27 and p57 protein levels were expressed as the ratio between their arbitrary densitometric units (ADU) and b-actin. Densitometry analyses were performed using the ImageQuant 5.2 software (GE Healthcare). (**D**) Expression profile of *Cdkn1c* gene measured by quantitative real-time PCR at different time points during neuronal differentiation of wild-type and *sik1^flpflp^* ES cells.

In order to visualize the temporal regulation of p57^Kip2^ throughout the differentiation process, we first evaluated the transcript levels of *Cdkn1c* gene in undifferentiated ES cells, in differentiating EBs and in fully differentiated cardiomyocytes, in both the wild-type and *sik1^flp/flp^* cells. We also investigated the expression levels of the two other members of the KIP family, *Cdkn1a* (p21) and *Cdkn1b* (p27), during cardiomyocyte differentiation. As shown in figure 6A, p57^Kip2^ was expressed at very low levels in undifferentiated wild-type ES cells and in day 1–5 differentiating EBs. From day 5 onward, p57^Kip2^ expression increased, and its expression correlated to the appearance of beating EBs. It is thus tempting to hypothesize that p57^Kip2^ is upregulated to progressively withdraw cells from the cell cycle and direct them toward differentiation. In *sik1^flp/flp^* cells, the expression levels of p57^Kip2^ were strongly downregulated throughout differentiation, being barely detectable (Fig. 6A). The levels of the two other members of the KIP family, p21 and p27, in the *sik1^flp/flp^* cells were comparable to those seen in wild-type cells, although a reduction in *p21* was observed (Fig. 6A). We confirmed these results using Western immunoblotting (Fig. 6B). As shown by the relative quantitative analysis (Fig. 6C), the protein levels were in accordance with the mRNA levels.

To further prove the specific effect of sik1 deficiency in the cardiogenic program, we measured the temporal expression of p57^Kip2^ throughout neuronal differentiation of both wild-type and *sik1^flp/flp^* ES cells. As opposed to what we observed in cardiac differentiation, the temporal regulation of *cdkn1c* gene was not affected throughout the neuronal differentiation in *sik1^flp/flp^* population (Fig. 6D). These results are in line with our previous data showing that sik1 deficiency does not affect neuronal differentiation (Fig. 5).

### Sik1 Directly Regulates p57^Kip2^

p57^Kip2^ and other CDKIs have pivotal roles in the regulation of the transition between cell cycle and terminal differentiation in many cell types [24].

To prove that downregulation of p57^Kip2^ directly affect the cardiogenic program, we evaluated whether re- expression of p57^Kip2^ in *sik1^flp/flp^* cells might rescue the cardiogenic defect of these cells.

**Figure 7:**
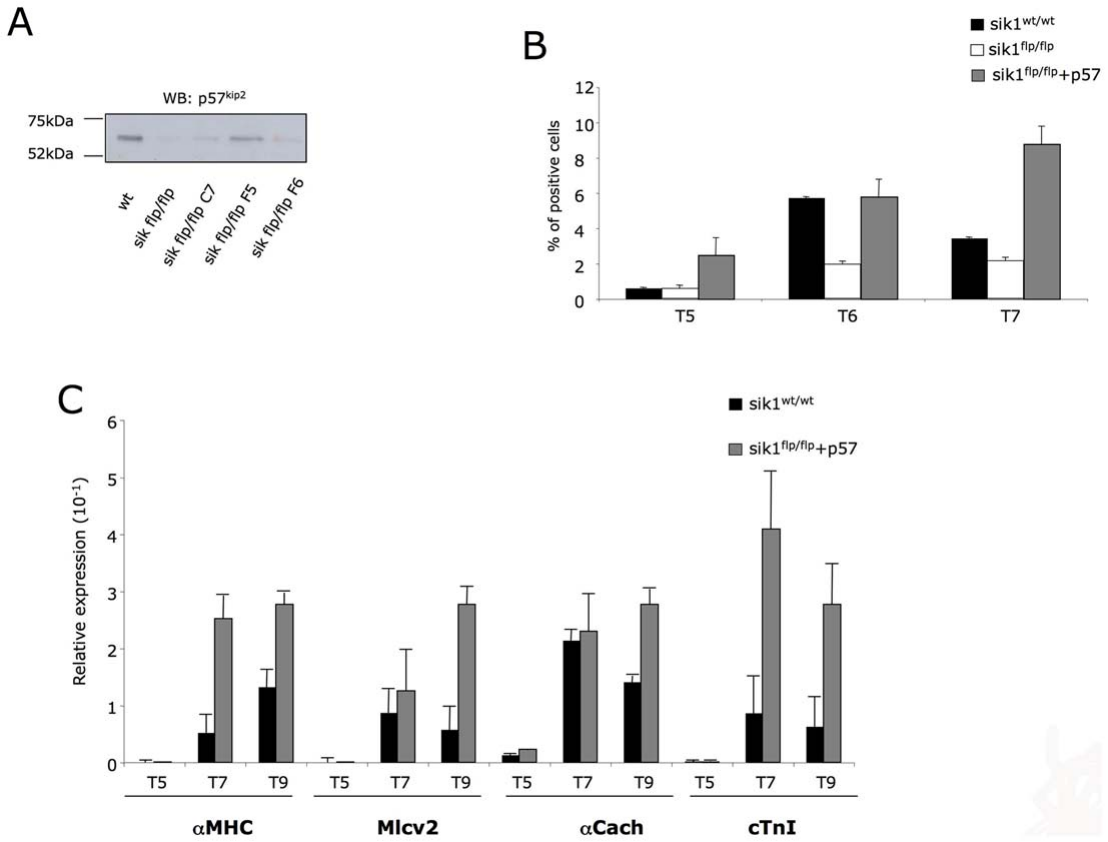
Over-expression of p57 in sik1^flp/fl^ cells. (A) Western blot analysis with the anti-p57 antibodies to detect the expression of transgene in three different clones in *sik1^flpflp^* ES cells. (B) Measurement of myosin positive cells in wild-type, sik1^flp/flp^ and sik1^flp/flp^ overexpressing p57 EBs by FACS, analyzed at days 0, 5, 6 and 7 of differentiation with the antibody MF20. Data represent results of one out of two independent experiments (C) Q-PCR analysis of terminal cardiac markers (*α-MyHC*, *Mlc2V*, *αCach* and c*TnI*).

To this end, we generated transgenic *sik1^flp/flp^* cells over-expressing p57^Kip2^. After selection of positive clones both by PCR (data not shown) and by western blot (Fig. 7A), we evaluated the effects of the p57^Kip2^ over-expression on cardiac differentiation. Remarkably, the over-expression of p57^Kip2^ in *sik1^flp/flp^* cells was able to fully rescue the cardiac phenotype, as shown by FACS and qPCR analysis. Upon forced expression of p57 the number of cardiomyocytes (MF-20 positive cells) significantly increased in the crucial time window (d5–d7) in *sik1^flp/flp^-derived EBs* (Fig. 7B). Expression data supported the FACS analysis (Fig. 7C), demonstrating that p57^Kip2^ over-expression has the ability to fully rescue the delayed phenotype observed in the *sik1^flp/flp^* cells.

Altogether, these findings demonstrated that sik1 regulated cardiomyocytes development via the p57^Kip2^ signaling.

## Discussion

*Sik1* (also known as SNF1LK, *sucrose nonfermented 1-like kinase*) encodes a serine/threonine kinase that belongs to a family of AMP-activated protein kinases (AMPK) involved in the regulation of metabolism during energy stress [25].

The expression pattern of *sik1* during embryogenesis suggests its involvement in cardiomyocyte differentiation and/or heart development. However, despite the significant research efforts toward the comprehension of the function of such a protein, the definition of its biological significance during cardiac development is still unknown.

We decided to investigate the role of *sik1* gene during cardiac differentiation using a gene trap ES cell line and revealed a novel role of *sik1 in* controlling the cell cycle progression of cardiomyoblasts.

Using the ES/EB model, we found that the absence of *sik1* produced a consistent delay of terminal cardiac differentiation and most remarkably, that this delay can be explained with the failure of the transcriptional regulation of the cyclin-dependent kinase inhibitor p57^Kip2^. It is worth noting here that the levels of p57^Kip2^ are very low in proliferating cells, and that differentiative signals result in its accumulation, which is necessary to withdraw cells from the cell cycle and to move them toward differentiation [8]. These data are in agreement with our observation that, during cardiac differentiation, the expression of p57^Kip2^ increases with the appearance of cardiomyocytes. Given that *sik1* deficiency completely suppressed the expression of p57^Kip2^, we speculated that defective signalling in the cell-cycle exit of these cells causes the delayed phenotype. The findings presented in this study indicate that sik1 signaling contributes to the cardiogenic program through the coordinated action of p57^Kip2^. Moreover, p57^Kip2^ over-expression experiment indicates that p57^Kip2^ is a downstream mediator of *sik1*, acting specifically in the cardiac differentiation process.

It is worth noting that the absence of *cdkn1c* transcriptional activation during differentiation of *sik1^flp/flp^* ES cells does not prevent the formation of mature cardiomyocytes. This might be explained by the expression of other members of the KIP/CIP family, as p21 and p27, that is not abolished during differentiation and which can likely compensate for the lack of *cdkn1c*. In addition, our results show that *sik1* specifically regulates the appearance of cardiac cells, since the timing of appearance of neurons is not affected by *sik1* deficiency. Our findings clearly shown that *sik1* is one of the regulators in the cascade of events leading to cardiomyogenesis through the modulation of p57^Kip2^ expression.

Notably, the balance between proliferation and terminal differentiation is a critical point in organ development [26], especially for the heart that is the first organ to form in the embryo [27]. Indeed, cardiac growth and chamber maturation are regulated by temporally and spatially controlled processes, resulting in a precise regulation of cell cycle exit and cell differentiation of cardiac cells. The ability of these cells to undergo cell division decreases progressively during heart development, such that adult myocytes cannot divide [7]. In this respect, cell cycle regulatory proteins are essential in orchestrating this process.

Although the identities of signals involved in cardiac specification are far to be fully elucidated, p57^Kip2^ is one of the intracellular candidates initially expressed in the myocardium at E10.5 [8]. Embryos lacking p57^Kip2^ exhibit hyperplasia in several organs and delayed differentiation, probably due to failure to exit from the cell cycle in a timely fashion. Surprisingly, cardiac defects have not been reported in p57^Kip2^ deficient mice [28], suggesting that other regulators are likely to be involved in a cooperative way with p57^Kip2^ in the cardiac cell cycle withdrawal and terminal differentiation. The gene profiling study performed on *sik1* deficient ES cells revealed that some of the transcriptional affected genes are expressed in the heart or involved in cardiac defects. This data suggests that *sik1* provide a critical growth signal for cardiomyoblasts, acting not only on *cdkn1c* gene, to promote cell cycle exit, but also on other genes involved in cardiomyocyte differentiation and/or heart development.

The observation that sik1 plays an important role in muscle function as HDAC5 kinase raises interesting questions regarding the possible link between HDAC5 and cell cycle in ES cells. HDAC5 belongs to class II HDAC enzymes and is highly enriched in muscles, heart and brain, as HDAC9 [29]. Although transgenic mice lacking either HDAC5 or HDAC9 are viable, mice lacking both HDAC5 and 9 show a propensity for lethal ventricular septal defects and thin-walled myocardium, which tipically arise from abnormalities in growth and maturation of cardiomyocytes [30].

The development of cardiac defects in these double KO mice probably results from a precocious differentiation and cell cycle withdrawal of cardiomyocytes, causing hypocellularity of the myocardium. Is sik1 involved in this intrigued mechanism? Our results shed light on the role of sik1 in cardiomyogenesis and, although further investigation is required to solve this issue, our data provide a new piece of puzzle in the understanding of the molecular mechanisms regulating cardiomyocyte cell cycle, which is of vital importance for future clinical approach.

To our knowledge, our manuscript provides the first evidence that sik1 via p57^Kip2^ plays a central role in the time clock mechanism that regulates cardiac cell proliferation and differentiation.

Worth noting, mouse *sik1* maps on mouse chromosome 17 in a region that is syntenic with human chromosome 21q22.3, where the human ortholog is located. It is well known that trisomy of 21 leads to Down syndrome, the most common genetic cause of mental retardation in the human population. Notably, forty percent of newborns with Down syndrome have congenital heart defects (CHD), of which the most frequent are atrioventricular septal defects [31]. Molecular studies of rare individuals with CHDs and partial duplications of chromosome 21 have established a candidate region of 5 Mb in which SIK is localized [32]. Given that *sik1* is specifically involved in cardiomyogenesis, we can speculate that this gene can play a role in CHDs observed in Down syndrome patients, opening new avenues in the understanding of the biological pathways involved in this severe disorder.

## Materials and Methods

### Ethics Statement

The experiments with murine cell lines were performed in compliance with the institutional guidelines (Italian Legislative Decree N° 120/92 (published on February 18th 1992) being the official Italian publication of the European Directive N° 88/320/CEE and 90/18/CEE concerning matters of inspections and verifications of Good Laboratory Practices). The parental cell line E14Tg2a.4 was obtained by Baygenomics, previously described in Brennan J, Skarnes WC. [33]. The generation of sik1wt/flp cell line was described in Cobellis, G et al. [14] and the sik1^flp/flp^ and HAsik1 cell lines were obtained by further modifications of sik1^+/flp^ cell line described in this report

### Plasmids

rSik1 coding was obtained digesting the plasmid pGFPC-rSik1 BamHI/EcoRI and cloned into the expression vector pcDNA3X(+)HA (Sigma-Aldrich). The *sik1* fragment encoding the amino acids 1–249 was amplified from ES cells cDNA with the primers sik1HAfor 5′-CTCGAGATGGTGATCATGTCGGAGT-3′ and sik1HArev 5′-TCTAGATCACTAAGGGTTCTTCTTCTTTGGT-3′ containing the XhoI and XbaI sites, respectively. The PCR product was subcloned in pCR-Blunt II-TOPO vector (Invitrogen), digested XhoI/XbaI from the TOPO Blunt II and cloned into pcDNA3X(+)HA.

The plasmid pGFPC-rSik1 was a kind gift of Dr. Hiroshi Takemori.

### Cell Culture

The feeder-independent mouse ES cell lines E14 Tg2A.4 (wild-type, *sik1* ^wt/flp^, *sik1* ^flp/flp^ and *sik1* ^flp/flp^ + HAsik1) were grown in DMEM supplemented with 15% (vol vol) fetal bovin serum, 2 mM L-glutamine, 1 mM MEM sodium pyruvate, 0.1 mM 2-mercaptoethanol (all from Invitrogen), 1,000 units/ml leukemia inhibitory factor (Esgro, Chemicon). HEK293T cells were maintained in DMEM (Invitrogen) containing 10% of fetal bovine serum (Hyclone).

### Production and Identification of Homozygous Mutant ES Cells

In order to obtain homozygous *sik1^flp/fl^* ^p^ ES cells, heterozygous *sik1^wt/flp^* ES cells were cultured in increasing concentrations of G418 (2 and 3 mg/ml) for 14 days as previously described [15]. After the selection, to identify homozygous *sik1^flp/fl^* ^p^ ES cells, 96 surviving clones were analyzed by RT-PCR using the following primers: sik1UP (5′-TCGGTGTGGTGCTGTACGT-3′), sik1LW (5′-ACTGCTGGGGGAGATGGAT-3′) and L232 (5′-GATGTGCTGCAAGGCGATTA-3′), as depicted in figure 1.

### Production of sik1^flp/flp^ + HAsik1 and p57 ES Cells

The sik1^flp/flp^ ES cell line was electroporated with 4 µg plasmid vector Pallino β-actin using the Nucleofector II (Amaxa biosystem). This plasmid vector contains the HAsik1 cDNA, driven by the β-actin promoter, and the puromycin resistance gene, driven by the phospho-glycerokinase promoter. The plasmid used to introduce p57 contains the p57 cDNA driven by PGK promoter. The stable selection marker is puromycin. In order to identify HAsik1 or p57 overexpressing ES cells, two weeks after selection with 1.2 µg/mL puromycin, the resistant colonies were screened by RT-PCR using the primers sik1UP and sik1LW for HAsik1 clones and with vector up and p57low for the p57 clones.

### ES Cells Differentiation

ES cells were differentiated by EB formation using the “hanging drop” method as previously described [13], [34]. The cardiomyocyte differentiation medium consists of the ES cells medium depleted of LIF. The neuronal and glial differentiation medium consists of the Knockout medium (Invitrogen) supplemented with 15% Knockout Serum SR, 2 mM L-glutamine, 1 mM sodium pyruvate, 0.1 mM 2-mercaptoethanol (all from Invitrogen) and depleted of LIF.

### Cell Proliferation Assay

Wild type and *sik1^flp/flp^* ES cells (5×10^4^ cells) were subjected to a pulse with 5-bromo-2′-deoxyuridine (BrdU) (Sigma, UK) for 14 hours at 37°C in gelatinized Chamber slide (Nunc) After that, the cells were washed with PBS (Invitrogen) then fixed for 10 minutes in 4% paraformaldehyde. The fixed cells were incubated with 50 mM Glycine and then permibilized with 50 mM NaOH for 10 seconds, washed in PBS and blocked in 5% goat serum/0,1% NP-40/PBS for 30 minutes at room temperature. BrdU incorporation into DNA was detected with a monoclonal antibody anti BrdU G3–G4 (Sigma-Aldrich) diluted 1∶500 at room temperature for 1 hour. Goat-anti mouse conjugated with FITC (Amersham) was used as secondary antiboby for immunofluorescence detection. Nuclei were counterstaining with DAPI and visualized with a fluorescent microscope. Images were taken with a digital camera (Zeiss Axiocam 1300×1030 pixel) mounted on a Axioplan 2 microscope (Carl Zeiss Inc.) with 60x NA 1.40 objectives (Carl Zeiss Inc.) at room temperature using AxioVision software (Carl Zeiss Inc.).

### Cell Cycle Analysis

Cellular DNA content was determined by staining cells with propidium iodide and analyzed them on a FACS instrument (Becton-Dickinson). ES cells were trypsinized, washed with PBS and resuspended in 500 µl of staining solution containing Na-citrate 0,1% (p/v), Nonidet P-40 0,1% (v/v), 10 µg/ml RNase and propidium iodide 50 µg/ml for 30 minutes. Cells were analyzed by FACS, and the proportion of cells in G0/G1, S, and G2/M phases was estimated by the Modfit cell-cycle analysis software.

### FACS Analysis

Flow cytometry was performed according to Hao J et al. [35]. Briefly, EBs were dissociated into single cell suspensions after trypsinization (2×10^6^ cells). Following a wash with 10%FBS/DMEM, cells were permeabilized with 0.05% saponin/PBS buffer for 20 minutes on ice. Cells were then stained with an antibody against sarcomeric myosin (MF-20 or cTnT antibodies, both from Hybridoma bank; dilution 1∶100 in 10%FBS/DMEM) for 1 hour. Following washes with 10%FBS/DMEM, cells were incubated with an anti-mouse secondary antibody conjugated to FITC (dilution 1∶100 in 10%FBS/DMEM) for 30 minutes in the dark. After additional washes (2x) in 10%FBS/DMEM, cells were resuspended in 500 µl 10%FBS/DMEM and analyzed on the FACS instrument (Becton-Dickinson).

The costaining with anti-BrdU-PE antibody was performed incubating the cells with bromodeoxiuridine (Roche) for 6 hr. After that, we proceeded with the anti-cTnI staining as previously described; then, cells were fixed with Cytofix-Perm solution (Becton Dickinson) for 20′ on ice and treated with DNAseI (Quiagen) to expose the DNA (1 hr at 37°C). Treated cells were incubated with anti-BrdU-PE (BD) for 30′ in the dark and analyzed on cytofluoremeter after extensive washes.

The number of labeled cells were determined with a FACSCalibur and analyzed by FlowJo software.

### Immunofluorescence

HEK293T cells were cultured on chamber slides (Lab-Tek II Chamber Slide w/Cover RS Glass Slide Sterile, Nunc) and transfected with the vectors pcDNA3X (+) HA-sik1 (1–249) and pcDNA3X (+) HA-rSik1 using the PolyFect Transfection Reagent (Qiagen). After 48 hours of transfection, cells were washed with PBS (Invitrogen), and fixed for 10 minutes in 4% paraformaldehyde. After extensive washing in PBS, the fixed cells were incubated with 10% of Bovine Serum Albumin and 0.1% Tween 20 diluted in PBS (Invitrogen) for 1 hour at room temperature, and then incubated with an anti-HA antibody (dilution 1∶250; Sigma-Aldrich) for 2 hours at room temperature. After extensive washing, the antigen-antibody complexes were visualized with anti-rabbit IgG-FITC conjugate IgG (dilution 1∶100; Amersham Biosciences) for 1 hour at room temperature. Nuclei were counterstained with DAPI. EB-derived neurons were plated into gelatin-coated 100 mm dishes (Nunc), washed with PBS (Invitrogen) and fixed for 10 minutes in 4% paraformaldehyde. The fixed EBs were incubated with 10% of Normal Goat Serum (Dako), 1% of Bovine Serum Albumine, 0.1% Triton X-100 diluted in PBS for 30 minutes at room temperature, and then reacted with an anti-βIII tubulin antibody (dilution 1∶400; Sigma-Aldrich) for 2 hours at room temperature. The antigen-antibody complexes were visualized with rabbit anti-mouse IgG-FITC conjugate IgG (dilution 1∶35; Dako) for 1 hour at room temperature. Nuclei were counterstained with DAPI. Images were taken through a digital camera (Zeiss Axiocam 1300×1030 pixel) mounted on an Axioplan 2 microscope (Carl Zeiss) with a 60x NA 1.40 lens (Carl Zeiss) at room temperature using AxioVision software (Carl Zeiss). For MF20 experiment, the fixation was performed with methanol: acetone (7∶3).

### Immunoprecipitation and Kinase Assay

Cells (2×10^6^) plated on a 10-cm dish were transformed with 6 µg of expression plasmids for FLAG-tagged wild type and mutant sik1 by using 20 µl of LipofectAMINE 2000 (Invitrogen). After 36 h of incubation, cells were lysed in 0.7 ml of lysis buffer. The FLAG-tagged sik proteins were immunoprecipitated with an anti- FLAG M2 affinity Gel (SIGMA). Aliquots of purified FLAG-SIK1 were then subjected to western blot analyses with the anti-FLAG-HRP antibodies and to *in vitro* kinase assays. Purified SIKs were incubated in 40 mM MOPS/NaOH pH 7.0, 1 mM EDTA with 0.1 mM of AMARA substrate (Enzo Life Sciences) in the presence of 25 mM MgAc and 0.5 mCi (18.5 kBq) of [γ-32P]-ATP at 30°C for 30 min. The kinase reaction was stopped by adding 5 ml of 3% phosphoric acid. An aliquot was transferred to P81 filters (Whatman) and after extensive washes in 75 mM phosphoric acid read in a scintillation counter.

### RNA Preparation, RT-PCR and Q-PCR

Total RNAs from either undifferentiated ES cells or differentiating EBs were extracted using RNeasy Miny Kit (Qiagen). First strand cDNA was synthesized from 1 µg of total RNA using QuantiTect Reverse Transcription kit (Qiagen). RT-PCR was performed on cDNA synthesized from wild-type and sik1^flp/flp^ + HAsik1 ES cells using LA-TAQ (TAKARA). sik1UP and sik1LW primers were used to amplify the sik1 transcript and GAPDH as an internal control. Amplification products were analyzed on ethidium bromide-stained 1% agarose gels and visualized at Gel Doc 2000 (Bio-Rad) using Quantity One software. Q-PCR was performed on the Light Cycler 480 real-time PCR system (ROCHE) using SYBR Green (Light Cycler 480 SYBR Green I master, ROCHE). Each sample was amplified in triplicate. The primers used are available on request.

### Microarray Hybridization and Analysis

Transcriptome analysis was performed comparing total RNA extracted from wild-type and mutant homozygous ES cells. The labeling of the RNAs, the hybridization on Affymetrix GeneChip MOE 430A 2.0 and analysis were performed by the Boston University Microarray Resource. The array data analysis was performed using the RMA algorithm.

The array experiments were submitted to MIAMEXPRESS and the assigned accession number is E-MEXP-1646.

### Detection of Functional DNA Motifs

The following bioinformatics approach was used to detect the presence of eventual functional DNA motifs regulating the expression of the genes found to be differentially expressed.

Affymetrix probeset IDs were used to retrieve a list of corresponding Ensembl transcript IDs from the Ensembl database (release 56, based on Mus Musculus NCBIM37). Transcript identifiers were then used to obtain sequences located within 1 kb upstream of each transcription strating site (TSS) (http://rsat.scmbb.ulb.ac.be/rsat/). Sequences were masked for the presence of repeats and coding sequences, as well as redundant sequences due to alternative transcripts were also avoided.

Sequences obtained were finally scanned for the presence of specific functional DNA motifs by using the Clover algorithm and the library of motifs provided by TRANSFAC® Professional 2009.2. For each sequence in both datasets, a P value was estimated as probability of obtaining a raw score of same size or greater merely by chance, using a shuffled version of the target sequence as control and a very stringent pvalue cut-off of 0.001.

### Western Blotting Analysis

ES cells and EB lysates were prepared in RIPA lysis buffer, separated by 10% SDS/PAGE and transferred onto PVDF membranes. Western blots were performed using antibodies against β-Galactosidase (dilution 1∶2000; Chemicon), p21 (dilution 1∶200; Abcam), p27 (M-197) (dilution 1∶200; Santa Cruz), p57 (H-91) (dilution 1∶200; Santa Cruz) and actin (dilution 1∶10000; Sigma-Aldrich). Detection of proteins was accomplished using Horseradish-peroxidase-conjugated secondary antibodies and enhanced chemioluminescence (ECL) purchased from Amersham. Scanning densitometry was performed using the ImageQuant software (Molecular Dynamics).

## Supporting Information

Table S1 Genes up-regulated in *sik1^flp/flp^* ES cells, FDR<5%.
(0.06 MB XLS)

Table S2 Genes down-regulated in *sik1^flp/flp^* ES cells, FDR<5%.
(0.07 MB XLS)

Table S3 Gene Ontology analysis on the gene-set transcriptionally affected in *sik1^flp/flp^* ES cells.
(0.04 MB DOC)

Table S4 Detection of functional DNA binding sites on genes differentially expressed between wt and *sik1^flp/flp^* ES.
(0.02 MB XLS)

Table S5 Transcriptionally affected genes by sik1 deficency expressed in heart or causing cardiac defects.
(0.12 MB DOC)
