# Supplementary material for: Lack of Sik1 in Mouse Embryonic Stem Cells Impairs Cardiomyogenesis by Down-Regulating the Cyclin-Dependent Kinase Inhibitor p57kip2
Source: PLoS One. 2010 Feb 3;5(2):e9029. doi: 10.1371/journal.pone.0009029 (PMC2815785; doi:10.1371/journal.pone.0009029)
Supplement: Table S3 — Gene Ontology analysis on the gene-set transcriptionally affected in sik1flp/flp ES cells. (0.04 MB DOC) [file pone.0009029.s003.doc]

**Table S3** Gene Ontology analysis on the gene-set transcriptionally affected in sik1^flp/flp^ ES cells.

| **GO Biological Process categories** | **Count** | **%** | **P-Value** | **Fold Enrichment** | **FDR** |
| --- | --- | --- | --- | --- | --- |
| steroid metabolic process | 12 | 2.6 | 5.60E-04 | 3.6 | 1.1 |
| calcium ion transport | 10 | 2.2 | 1.20E-03 | 3.8 | 2.2 |
| ion transport | 31 | 6.8 | 1.80E-03 | 1.8 | 3.3 |
| di-, tri-valent inorganic cation transport | 11 | 2.4 | 2.00E-03 | 3.3 | 3.8 |
