# Supplementary material for: Lack of Sik1 in Mouse Embryonic Stem Cells Impairs Cardiomyogenesis by Down-Regulating the Cyclin-Dependent Kinase Inhibitor p57kip2
Source: PLoS One. 2010 Feb 3;5(2):e9029. doi: 10.1371/journal.pone.0009029 (PMC2815785; doi:10.1371/journal.pone.0009029)
Supplement: Table S5 — Transcriptionally affected genes by sik1 deficency expressed in heart or causing cardiac defects. (0.12 MB DOC) [file pone.0009029.s005.doc]

**Table S5** Genes Transcriptionally affected by sik1 deficency expressed in heart or causing cardiac defects. The genes with heart expression were detected by RNA *in situ* hybridization or by immunoistochemestry (*****).

| **Genes detected in heart** | |
| --- | --- |
|  |  |
| **Gene description** | **Gene symbol** |
|  |  |
| RIKEN cDNA 4631426J05 gene | 4631426J05Rik |
| actin, alpha, cardiac | Actc1 |
| apolipoprotein B editing complex 2 | Apobec2 |
| ATPase, Ca++ transporting, cardiac muscle, fast twitch 1 | Atp2a1 |
| CAP, adenylate cyclase-associated protein, 2 (yeast) | Cap2 |
| cyclin-dependent kinase inhibitor 1C (P57) | Cdkn1c |
| cholinergic receptor, nicotinic, alpha polypeptide 7 | Chrna7 |
| carboxypeptidase D | Cpd |
| cytochrome P450, family 26, subfamily a, polypeptide 1 | Cyp26a1 |
| dickkopf homolog 2 (Xenopus laevis) | Dkk2 |
| dual specificity phosphatase 9 | Dusp9 |
| forkhead box M1 | Foxm1 |
| gelsolin | Gsn |
| hepatocyte growth factor | Hgf |
| inhibin beta-A | Inhba |
| junction adhesion molecule 2 | Jam2 |
| kallikrein 8 | Klk8 |
| LIM and cysteine-rich domains 1 | Lmcd1 |
| mesenchyme homeobox 1 | Meox1 |
| neurogenin 1 | Neurog1 |
| noggin | Nog |
| phosphatidylinositol-4-phosphate 5-kinase, type 1 alpha | Pip5k1a |
| paired-like homeodomain transcription factor 2 | Pitx2 |
| phospholipase C, beta 1 | Plcb1 |
| secretin | Sct |
| synaptotagmin 10 | Syt10 |
| T-box 3 | Tbx3 |
| transforming growth factor, beta 2 | Tgfb2 |
| wingless-related MMTV integration site 8A | Wnt8a |
| dystrobrevin alpha ***** | Dtna |
| fibroblast growth factor 8 ***** | Fgf8 |
| glial cell line derived neurotrophic factor family receptor alpha 2 ***** | Gfra2 |
| matrix metalloproteinase 9 ***** | Mmp9 |

|  |  |
| --- | --- |
| **Genes involved in cardiac defects** | |
|  |  |
| **Gene description** | **Gene symbol** |
|  |  |
| ATP-binding cassette, sub-family A (ABC1), member 1 | Abca1 |
| a disintegrin and metalloproteinase domain 15 (metargidin) | Adam15 |
| ankyrin 1, erythroid | Ank1 |
| apelin | Apln |
| caveolin 3 | Cav3 |
| CD8 antigen, alpha chain | Cd8a |
| procollagen, type I, alpha 1 | Col1a1 |
| colony stimulating factor 1 (macrophage) | Csf1 |
| chemokine (C-X-C motif) ligand 16 | Cxcl16 |
| ephrin B2 | Efnb2 |
| EGL nine homolog 3 (C. elegans) | Egln3 |
| ectonucleotide pyrophosphatase/phosphodiesterase 2 | Enpp2 |
| coagulation factor III | F3 |
| FXYD domain-containing ion transport regulator 1 | Fxyd1 |
| hyaluronan synthase 2 | Has2 |
| histone 1, H1c | Hist1h1c |
| Hermansky-Pudlak syndrome 1 homolog (human) | Hps1 |
| hydroxysteroid (17-beta) dehydrogenase 7 | Hsd17b7 |
| 5-hydroxytryptamine (serotonin) receptor 1B | Htr1b |
| integrin beta 4 | Itgb4 |
| klotho | Kl |
| leptin receptor | Lepr |
| melanocortin 2 receptor | Mc2r |
| Notch gene homolog 4 (Drosophila) | Notch4 |
| nuclear receptor subfamily 1, group H, member 3 | Nr1h3 |
| pre B-cell leukemia transcription factor 1 | Pbx1 |
| pleiomorphic adenoma gene-like 1 | Plagl1 |
| presenilin 2 | Psen2 |
| prostaglandin E synthase | Ptges |
| protein tyrosine phosphatase, receptor type, B | Ptprb |
| selectin, endothelial cell | Sele |
| serine (or cysteine) proteinase inhibitor, clade E, member 1 | Serpine1 |
| solute carrier family 11 (proton-coupled divalent metal ion transporters), member 2 | Slc11a2 |
| brachyury | T |
| transient receptor potential cation channel, subfamily C, member 4 | Trpc4 |
